# Polymorphisms in *GC* and *NADSYN1* Genes are associated with vitamin D status and metabolic profile in Non-diabetic adults

**DOI:** 10.1186/1472-6823-13-36

**Published:** 2013-09-29

**Authors:** Lydia Foucan, Fritz-Line Vélayoudom-Céphise, Laurent Larifla, Christophe Armand, Jacqueline Deloumeaux, Cedric Fagour, Jean Plumasseau, Marie-Line Portlis, Longjian Liu, Fabrice Bonnet, Jacques Ducros

**Affiliations:** 1 Research group Clinical Epidemiology and MedicineResearch group, University of Antilles and Guyane, Guyane, France; 2 Department of Public Health and Medical Information, University Hospital of Pointe-à-Pitre, Guadeloupe, France; 3 Diabetology Unit, University Hospital of Pointe-à-Pitre, Guadeloupe, France; 4 Health Centre, AGREXAM, Les Abymes, Guadeloupe, France; 5 Clinical Genetic Unit, University Hospital of Pointe-à-Pitre, Guadeloupe, France; 6 Department of Epidemiology and Biostatistics, Drexel University School of Public Health, Philadelphia, PA, USA; 7 Endocrinology-Diabetology and nutrition Unit, University Hospital South of Rennes, Rennes, France; 8 Nephrology Unit, University Hospital of Pointe-à-Pitre, Guadeloupe, France; 9 Département de Santé Publique, CHU de Pointe-à-Pitre, 97159 Pointe-à-Pitre, Guadeloupe, France

**Keywords:** Dyslipidemia, Overweight, Vitamin D, NAD synthetase 1, NADSYN, Group specific component, GC

## Abstract

**Background:**

Our aim was to assess the associations between vitamin D (vitD) status, metabolic profile and polymorphisms in genes involved in the transport (*Group-Component*: *GC*) and the hydroxylation (*NAD synthetase 1*: *NADSYN1)* of 25 hydroxyvitamin D (25(OH)D) in non-diabetic individuals.

**Methods:**

We conducted a cross-sectional study with 323 individuals recruited from the Health Center of Guadeloupe, France. The rs2282679 T > G and rs2298849 T > C in *GC* and rs12785878 G > T in *NADSYN1* were genotyped.

**Results:**

Mean age was 46(range 18–86) years. 57% of participants had vitD insufficiency, 8% had vitD deficiency, 61% were overweight and 58% had dyslipidemia. A higher frequency of overweight was noted in women carrying rs2298849T allele *v* CC carriers (71% *v* 50%; *P* = 0.035). The rs2282679G allele was associated with increased risks of vitD deficiency and vitD insufficiency (OR =3.53, *P* = 0.008, OR = 2.34, *P* = 0.02 respectively). The rs2298849 TT genotype was associated with vitD deficiency and overweight (OR =3.4, *P* = 0.004 and OR = 1.76, *P* = 0.04 respectively) and the rs12785878 GG genotype with vitD insufficiency and dyslipidemia (OR = 1.80, *P* = 0.01 and OR = 1.72, *P* = 0.03 respectively). Based on the number of risk alleles for rs2282679 and rs12785878 combined, a genotype score of 3 (vs. 0–1) was associated with a 5.5 ng/mL average reduction in serum 25(OH)D levels (*P* = 0.001).

**Conclusions:**

The *GC* and *NADSYN1* genes are associated with the vitamin D status and might contribute to dyslipidemia and overweight independently of 25(OH)D levels.

## Background

The storage form of vitamin D, 25-hydroxyvitamin D (25(OH)D), measured in blood circulation is a marker of vitamin D status. Low 25(OH)D has been associated with increased risk of cardio-metabolic diseases, including obesity [1], dyslipidemia [2], type 2 diabetes [3], and cardiovascular complications [2,4]. However, genetic factors may also have effects on these diseases. In particular, single-nucleotide polymorphisms (SNPs) in genes of the vitamin D pathway that are involved in the transport or the hydroxylation of 25(OH)D may be associated with the cardio-metabolic risk.

The 25(OH)D is transported in the circulation, mainly bound to its specific vitamin D-binding protein, also named the GC-group component (GC). As the VDR gene [5], polymorphisms in the *GC* gene were previously reported to be associated with plasma glucose levels [6], fasting plasma insulin levels [7] and the percentage of fat mass in Caucasian nuclear families [8].

In previous studies of 25(OH)D, variants of genes involved in vitamin D transport (*GC*), hydroxylation/dehydroxylation (*CYP2R1* and *CYP24A1*), and *NADSYN1* were reported to modulate vitamin D status [9-11]. The *NADSYN1* gene encodes nicotinamide adenine dinucleotide synthetase 1 (NADSYN1), which is one of the glutamine-dependent enzymes involved in cholesterol synthesis and favors the production of nicotinamide adenine dinucleotide (NAD+), the main coenzyme required for energy production and lipid synthesis [12]. Nevertheless, studies of the association between overweight or dyslipidemia and polymorphisms in these genes are scarce. A recent study performed in an African-American cohort found associations between SNPs in the *GC* and *CYP27B1* genes and 25(OH)D with a significant relation with the degree of African ancestry as assessed using skin color gradation [13].

In the French Caribbean island of Guadeloupe, which has 400,000 inhabitants, about 80% of the population is of African descent and these individuals are at risk of vitamin D insufficiency and obesity [14,15].

Our aim was to assess the associations between vitamin D status, overweight, dyslipidemia and four SNPs of the *GC*, *CYP27B1*, and *NADSYN1* genes in non-diabetic individuals with African ancestry.

## Methods

### Population study

The participants were recruited among individuals who had a clinical and biological examination at the referring Health Center of Guadeloupe. Data were collected during 2010 and 2011. For the assessment of vitamin D status, the physician included volunteers during sessions that were randomly planned. The determination of African ethnic background was based on self-report. Exclusion criteria included a history of kidney disease, diabetes, or inflammatory disease, pregnancy, and calcium or vitamin D replacement therapy. The study was approved by the inter-regional ethic committee (Sud-Ouest et Outre-Mer III, France). All patients gave their written informed consent to participate in this study.

### Data collection

Participants were interviewed by a physician using a standard questionnaire that provided information on age, sex and use of antihypertensive and/or anti-hyperlipidemic treatments.

Height and weight were measured with participants standing without shoes and lightly clothed. Body mass index (BMI) was calculated as weight/height (kg/m^2^). Waist circumference (cm) was measured above the iliac crests and below the lowest rib margin at minimal respiration with participants standing. The measurements were performed by trained nurses. Systolic and diastolic blood pressures were assessed using automated monitors after resting for at least 5 min. The retained values were the average of two readings (left and right arms).

### Laboratory measurements

Blood samples were obtained after an overnight fast. The biochemical analyses were performed using the same methods for the whole study sample. Glycaemia was assessed using the glucose oxidase method. Cholesterol, high-density lipoprotein/cholesterol, and triglyceride levels were measured enzymatically. Plasma concentrations of 25(OH)D were measured via a chemiluminescence assay (DiaSorin SA, Antony, France), which includes 25(OH)D2 and 25(OH)D3. In this Health Center, only single measurements were available for all the parameters.

### Genotyping

Genotyping was performed in 323 Afro-Caribbean individuals for SNPs that were reported to be associated with vitamin D status in individuals of European descents (11) or in African-Americans (14): rs2282679 (intron 12) in *GC*, rs2298849 (intron 1) in *GC*, rs10877012 (5′ UTR) in CYP27B1, and rs12785878 (intronic region of NM_018161.4) in *NADSYN1*. DNA was extracted from peripheral blood samples by standard procedures. SNPs were genotyped by KBioscience Ltd using their own novel fluorescence-based competitive allele-specific PCR (KASPar) assay. Details of the method used can be found at http://www.kbioscience.co.uk/.

### Definition of clinical factors

*Obesity* was defined as a BMI ≥ 30 kg/m^2^ and *overweight* was defined as a BMI ≥ 25 kg/m^2^. *Abdominal obesity* was defined as a waist circumference > 102 cm in men or > 88 cm in women.

*Vitamin D insufficiency* was defined as a 25(OH)D level < 30 ng/mL and *vitamin D deficiency* as a 25(OH)D level < 20 ng/mL.

*Dyslipidemia* was defined as having one of the following measurements: high-density lipoprotein/cholesterol concentration < 40 mg/dL in men and < 50 mg/dL in women, triglyceride concentration ≥ 150 mg/dL, low-density lipoprotein/cholesterol concentration ≥ 130 mg/L, or the presence of a lipid-lowering treatment combined with a history of blood lipid abnormality [16].

### Statistical analyses

The chi-squared test and ANCOVA were used to test percentage and mean differences between groups. Serum 25(OH)D was log_10_ transformed to approach a normal distribution.

We examined the associations between SNPs, vitamin D deficiency, vitamin D insufficiency, dyslipidemia and overweight status. The logistic regression models were tested including each SNP alone with adjustment for age, sex, BMI, 25(OH)D levels, or dyslipidemia, according to the dependent variable. Adjusted odds ratios (ORs) and 95% confidence intervals (95% CIs) were estimated. The SNP effects were presented as ORs associated with the genotypes.

We used a genotype score approach to evaluate the combined effects of SNPs that were significantly associated with serum 25(OH)D. The genotype score is equal to the sum of the number of risk allele (25(OH)D lowering allele) in the SNPs. To evaluate the variation in 25(OH)D levels associated with the genotype score, we applied simple and multiple linear regression analysis methods using serum 25(OH)D level as the dependent variable, and genotype score as the independent variables. Adjustments were performed for age, sex and BMI. The genotype score effects were assessed by the values of regression coefficients (beta) corresponding to the non-standardized regression coefficients.

The IBM SPSS Statistics software version 21.0 was used for data analyses. All tests were two-sided and a *P* value < 0.05 was considered significant.

## Results

### Characteristics of the study population

Three hundred twenty-three non-diabetic individuals were included in the study. Among the participants, 187 (58%) were women. The mean age was 46 ± 12 years.

The characteristics of the population under study are shown in Table 1. Among the participants, 29% were obese, 61% were overweight, 42% had hypertension, 58% had dyslipidemia, 57% had vitamin D insufficiency and 8% had vitamin D deficiency. In the whole study population, the concentration of 25(OH)D ranged from 7 to55 ng/mL and the mean serum 25(OH)D was 29 ± 8 ng/mL.

**Table 1:** Characteristics of the study population

**Variables**	**N**	**All subjects**	**Men**		**Women**		***P***
Sex (F) (n (%))	**323**	136 (58)	136	__	187	__	
Age (years)	**323**	46 ± 12	136	41 ± 12	187	47 ± 12	0.42
Waist circumference (cm)	**307**	89 ± 13	130	89 ± 11	177	90 ± 14	0.40
Body mass index (Kg/m^2^)	**323**	27 ± 6	136	25 ± 4	187	29 ± 7	**<0.001**
Glycaemia (mmol/L)	**323**	4.6 ± 1.2	136	4.9 ± 1.6	187	4.5 ± 0.9	**0.003**
25(OH)D (ng/mL)	**323**	29 ± 8	136	30 ± 8	187	28 ± 7	**0.006**
Overweight (n (%))	**323**	198 (61)	136	71 (52)	187	127 (68)	**0.004**
Obesity (n (%))	**323**	94 (29)	136	23 (17)	187	71 (38)	**<0.001**
Abdominal obesity (n (%))	**307**	108 (35)	130	13 (10)	177	95 (54)	**<0.001**
Hypertension (n (%))	**323**	136 (42)	136	56 (41)	187	80 (43)	0.77
Dyslipidemia (n (%))	**319**	184 (58)	135	69 (51)	184	115 (63)	**0.04**
Vitamin D Insufficiency (n (%))	**323**	183 (57)	136	68 (50)	187	115 (62)	**0.04**

The data are presented as mean ± SD or number (%).

The prevalence of obesity and overweight status were significantly higher in women than in men (38% *v*17%, *P* < 0.001 and 68% *v* 52%, *P* = 0.004, respectively).

The genotype distributions in the study population were within the Hardy–Weinberg equilibrium for rs2282679 T > G (0% GG, 13% TG, 87% TT; *P* = 0.90), rs2298849 T > C (15% CC, 51% TC, 34% TT; *P* = 0.90) and rs12785878 G > T (5% TT, 33% GT, 62% GG; *P* = 0.90), but not for rs10877012 G > T (25% GT, 75% GG; *P* < 0.05). Consequently, the results for rs10877012 (*CYP27B1*) are not shown in this report.

### Distributions of 25(OH)D levels and metabolic parameters according to genotypes

The distributions of 25(OH)D levels and metabolic parameters according to genotypes of *GC* and *NADSYN1* genes are presented in Table 2.

**Table 2:** **Distribution of 25(OH)D levels and metabolic parameters according to genotypes of *GC *and *NADSYN1 *genes**

	**Genotypes**				**Dominant model**	**Recessive model**
**rs2282679 T > G *(GC)***	**GG**	**TG**	**TT**	P*		
(*N* = 323)	**0**	**43**	**280**			
25 (OH)D (ng/mL)	__	26.4 ± 8.3	29.0 ± 7.4	**0.03**	__	__
Vitamin D Insufficiency	__	72%	54%	**0.03**	__	__
Vitamin D Deficiency	__	19%	6%	**0.01**	__	__
Overweight	__	67%	60%	0.37	__	__
Obesity	__	35%	28%	0.37	__	__
Abdominal obesity	__	42%	34%	0.36	__	__
Dyslipidemia	__	44%	60%	0.05	__	__
**rs2298849 T > C *(GC)***	**CC**	**TC**	**TT**	P*	***P***	***P***
(*N* = 323)	**49**	**165**	**109**			
25 (OH)D (ng/mL)	28.3 ± 6.9	28.5 ± 7.7	29.0 ± 7.8	0.87	0.60	0.76
Vitamin D Insufficiency	53%	60%	53%	0.46	0.37	0.58
Vitamin D Deficiency	6%	5%	14%	**0.03**	**0.007**	0.50
Overweight	51%	61%	66%	0.20	0.21	0.11
Obesity	22%	32%	28%	0.46	0.85	0.26
Abdominal obesity	28%	39%	33%	0.32	0.37	0.27
Dyslipidemia	53%	63%	51%	0.13	0.11	0.48
**rs12785878 G > T**	**TT**	**GT**	**GG**	P*	***P***	***P***
**( *NADSYN1 *)**(*N* = 318)	**15**	**106**	**197**			
25 (OH)D (ng/mL)	27.7 ± 7.1	30.2 ± 8.3	27.8 ± 7.1	0.05	**0.03**	0.69
Vitamin D Insufficiency	53%	47%	62%	**0.04**	**0.01**	0.77
Vitamin D Deficiency	13%	5%	10%	0.25	0.23	0.46
Overweight	40%	62%	62%	0.22	0.60	0.08
Obesity	20%	33%	28%	0.50	0.85	0.40
Abdominal obesity	21%	39%	34%	0.37	0.37	0.17
Dyslipidemia	60%	49%	62%	0.08	**0.04**	0.83

The data are presented as mean (SD) or column percentage. Significant P values are presented in bold.

*P values for comparisons between genotypes.

For the rs2282679 SNP in the *GC* gene, lower values of serum vitamin D, higher frequencies of vitamin D insufficiency and of vitamin D deficiency were observed in carriers of the TG genotype than in the remaining individuals (*P* values from 0.03 to 0.01). The association of this SNP with dyslipidemia was nearly significant (*P* = 0.05).

Significant differences were noted in frequency of vitamin D deficiency according the rs2298849 genotypes with a higher frequency in carriers of the TT genotype than in the non-carriers (*P* = 0.007). For rs12785878, higher values for mean circulating 25(OH)D (*P* = 0.03) and lower frequency of vitamin D insufficiency (*P* = 0.01) and dyslipidemia (*P* = 0.04) were noted in carriers of the minor allele (TT/GT) compared with the remainder of the cohort.

Regarding the associations of the three SNPs studied with overweight or obesity according to sex, a higher frequency of overweight was noted in women carrying rs2298849T allele *vs* CC carriers (71% *v* 50%; *P* = 0.035)(Table 3). In men, no relationship was found between obesity or overweight and the three SNPs studied.

**Table 3:** Frequencies of overweight according to rs2298849 T > C genotypes in men and women

	**MEN**			**WOMEN**		
	**N = 136**			**N = 187**		
**Overweight**	**TT-TC**	**CC**	***P***	**TT-TC**	**CC**	***P***
	**n = 103**	**n = 23**		**n = 161**	**n = 26**	
**No**	44 (48)	11 (48)	0.99	47 (29)	13 (50)	**0.035**
**Yes**	59 (52)	12 (52)		114 (71)	13 (50)	

The data are presented number (column percentage).

### Logistic regression of vitamin D deficiency, vitamin D insufficiency, dyslipidemia and overweight

Table 4 presents the adjusted odds ratios (OR) for risk of vitamin D deficiency, vitamin D insufficiency, dyslipidemia and overweight according to the *GC* and the *NADSYN1* genotypes. For rs2282679, carrying the G allele increased the risk of vitamin D deficiency and of vitamin D insufficiency (adjusted OR = 3.53, *P* = 0.008 and adjusted OR = 2.34, *P* = 0.02 respectively) in comparison with the TT genotype and surprisingly with a decreased risk of dyslipidemia.The odds of vitamin D deficiency was significantly increased for rs2298849 TT carriers in comparison with the rs2298849C allele carriers (adjusted 3.40 = 1.46, *P* = 0.004) and that of vitamin D insufficiency was significantly increased for rs12785878 GG carriers (adjusted OR =1.80, *P* = 0.01) in comparison to T allele carriers. The rs12785878 was also associated with an increased risk of dyslipidemia.

**Table 4:** **Logistic regressions of vitamin D deficiency, vitamin D insufficiency, dyslipidemia and overweight for polymorphisms in *GC *and *NADSYN1***

	**Vitamin D**		**Vitamin D**		**Dyslipidemia**		**Overweight**	
	**deficiency**		**Insufficiency**					
	**Adjusted**	***P***	**Adjusted**	***P***	**Adjusted**	***P***	**Adjusted**	***P***
	**OR (95% CI)**		**OR (95% CI)**		**OR (95% CI)**		**OR (95% CI)**	
*GC***rs2282679** T > G								
TT	1		1		1		1	
TG- GG	3.53 (1.38 - 9.80)	**0.008**	2.34 (1.15 - 4.90)	**0.02**	0.41 (0.21 - 0.84)	**0.01**	1.60 (0.70 – 3.36)	0.20
*GC***rs2298849** T > C								
CC - TC	1		1		1		1	
TT	3.40 (1.46 - 7.89)	**0.004**	0.82 (0.51 - 1.31)	0.41	0.67 (0.40 - 1.11)	0.12	1.76 (1.04 - 2.98)	**0.04**
*NADSYN1***rs12785878** G > T								
TT - GT	1		1		1		1	
GG	1.72 (0.69 - 4.24)	0.54	1.80 (1.12 - 2.87)	**0.01**	1.72 (1.04 - 2.86)	**0.03**	1.02 (0.62 - 1.69)	0.93

*Vitamin D deficiency and vitamin D insufficiency*: adjustment for age, sex, BMI, dyslipidemia. *Dyslipidemia:* adjustment for age, sex, BMI, 25(OH)D levels.

*Overweight*: adjustment for age, sex, 25(OH)D levels, dyslipidemia. The adjusted odds ratios are provided, separately for each SNP. Significant P values are presented in bold.

### Serum 25(OH)D levels in relation to genotype score

**Figure 1:**
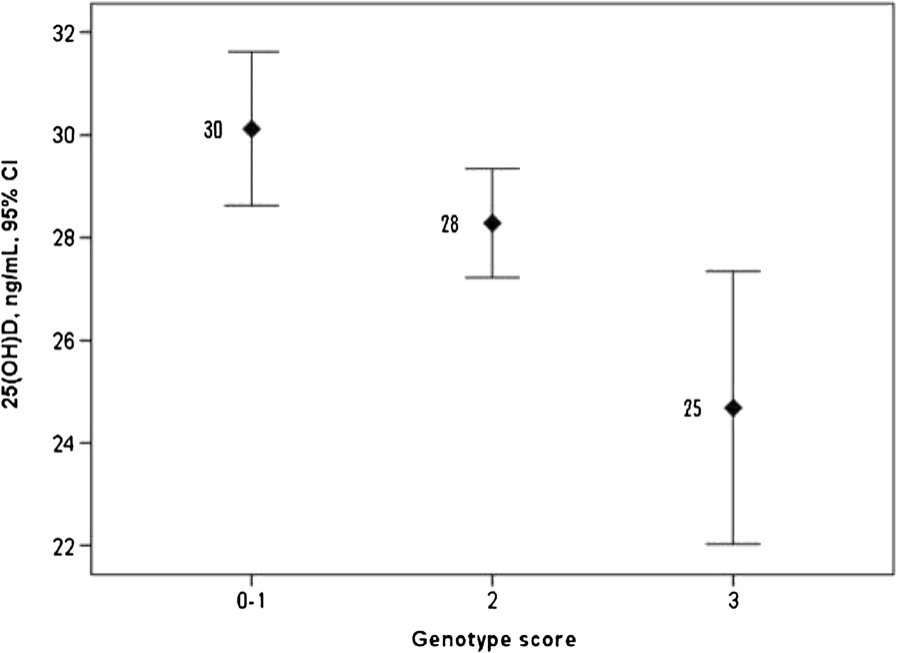
**Mean serum 25(OH)D levels and 95% confidence intervals according to genotype scores of two SNPs (rs2282679 T > G and rs2298849 T > C). *P*** **= 0.007.**

The risk alleles (25(OH)D lowering allele) were G for rs2282679, T for rs2298849 and G for rs12785878. The high risk alleles of rs2282679 and rs12785878 were combined and the range of the genotype score was 0–3. The genotype score was significantly related to 25(OH)D and mean serum 25(OH)D levels (95% CI) were 30.1 (28.6 – 31..6) ng/mL for score 0–1, 28.3 (27.2 – 29.3) ng/mL for score 2 and 24.7 (22.0 – 27.3) ng/mL for score 3, *P* = 0.007 (Figure 1).

Table 5 presents the results of the linear regressions between the genotype scores across the rs2282679 and rs12785878 SNPs and serum 25(OH)D levels. Genotype score of 0–1 was considered as referent. **In model 1** (1a, 1b, 1c) with the simple linear regression and considering each SNP alone, significant average change in 25(OH)D levels were noted for a rs2282679 genotype score of 2 (vs 1) (**model 1a**) and for a rs12785878 genotype score of 2 (vs 0–1) (**model 1c**). These both models accounted for 1% (R^2^ = 0.01) and 2% (R^2^ = 0.02), of the variation in 25(OH)D levels, respectively. In the multiple linear regression, the average change in 25(OH)D levels remained significant with multiple R^2^ of 0.06 for both models. The rs2298849 genotype score was not significantly associated with 25(OH)D levels. We also noted that BMI was not related to the genotype score of the three SNPs (rs2282679; *P* = 0.75, rs2298849; *P* = 0.65, rs12785878; *P* = 0.75, data not shown).

**Table 5:** **Linear regression: between serum 25-hydroxyvitamin D levels and genotype scores for rs2282679, rs2298849, rs12785878**

		**Simple linear regression**			Multiple linear regression**		
	**Genotype score**	Beta*	***P***	**R**^**2 **^**for the model**	Beta*	***P***	**R**^**2 **^**for the model**
***Model 1a*** risk allele **G** for **rs2282679** T > G	2 (vs 1)	−2.5	**0.04**	*0.01*	−2.6	**0.03**	*0.06*
***Model 1b*** risk allele **T** for **rs2298849** T > C	2 (vs 0–1)	0.70	0.43	*0.002*			
***Model 1c*** risk allele **G** for **rs12785878** G > T	2 (vs 0–1)	−1.5	**0.04**	*0.01*	−2.1	**0.02**	*0.06*
***Model 2***							
risk alleles **G**							
for **rs2282679** T > G and **rs12785878** G > T	2 (vs 0–1)	−1.8	**0.04**	*0.03*	−1.6	0.06	*0.08*
	3 (vs 0–1)	−5.4	**0.02**		−5.5	**0.001**	

*Model 1a, 1b* include genotype scores for each SNP alone. *Model 2* includes genotype scores for rs2282679 and rs12785878 combined.

Genotype score equals the sum of the number of risk alleles.*Beta: average change in serum 25(OH)D level (ng/mL) associated with the genotype. Regression: ** with adjustment for age, sex, BMI.

**In models 2,** and in the simple regression, progressive reductions in 25(OH)D levels were noted for score 2 and 3 (compared to 0–1): beta = −1.8 ng/mL (*P* = 0.04) and beta = −5.4 ng/mL (*P* = 0.02) respectively. In the multiple regression with adjustment for age, sex and BMI, the statistically significant average change in 25(OH)D levels for score of 3 (vs 0–1) was −5.5 ng/mL (*P* = 0.001) with R^2^ of 0.08. The combination of both genotype scores accounted for a 2% increase of R^2^. Conversely, this genotype score was not associated with BMI (data not shown).

## Discussion

In this study examining the relationship between vitamin D status and SNPs of the vitamin D-binding protein (*GC*) and *NADSYN1* genes in non-diabetic individuals with African ethnic background, we found significant associations between rs2282679 (*GC*), rs2298849 (*GC*), and rs12785878 (*NADSYN1*) and vitamin D status. Significant associations were also noted with overweight for rs2298849 and with dyslipidemia for rs12785878 and rs2282679.

In this study population living in a sunny climate, the prevalence of vitamin D insufficiency was 57% and that of vitamin D deficiency was 8%. These prevalence values were globally lower than that observed in African-American individuals living in countries with a temperate climate [14,17]. The effect of sunshine on vitamin D levels is widely recognized.

The vitamin D-binding protein, which is also known as the group-specific component (GC), is the main transporter of vitamin D [10]. The concentration of this serum glycoprotein influences 25(OH)D levels and modulates the rates of its bioavailability [18-20]. The *GC* gene is localized on chromosome 4 (4q12–q13), encodes a single-chain polypeptide that comprises 474 amino acid residues and belongs to the albumin family [10,21-23].

Our results regarding vitamin D corroborate previous reports which showed associations between vitamin D levels and rs2282679 and rs2298849 in the *GC* gene in African-Americans [13], rs2282679 in Caucasians in two genome wide association (GWA) studies [9,11] and rs2298849 in 496 healthy individuals [12]. In addition, the genotype distributions of the two *GC* SNPs in our African-Caribbean population were close to those observed in the study conducted among AfricanAmericans individuals [13] for rs2282679 (0.2% for the rare homozygote, 15.2% for the heterozygote and the, 84.5% for the frequent homozygote genotype) and for rs2298849 (16.4% for the rare homozygote, 46.2% for the heterozygote and 37.4% for the frequent homozygote genotype).

The effects of *CYP27B1 and NADSYN1* on circulating 25(OH)D were also reported in these studies [9,11] and confirmed in a recent study in Han Chinese children [24].Two other SNPs in the *GC* gene, rs4588 and rs7041, which have been more commonly studied, exhibited significant associations with the levels of 25(OH)D and 1,25(OH)2D in Hispanic- and African-Americans [25], in Caucasian women [26], and in young Canadian adults of East-Asian, European, and South-Asian ancestry [19].

The relationship between the *NADSYN1* gene and vitamin D status has been less studied. The *NADSYN1* gene is located on chromosome 11 (11q13.4), close to the dehydrocholesterol reductase (*DHCR7*) gene, which encodes 7-dehydrocholesterol reductase, an enzyme involved in the conversion of 7-dehydrocholesterol into cholesterol in human skin [27]. A recent GWA study revealed that variants near genes involved in cholesterol synthesis influence the vitamin D status [11]. The rs12785878 GG genotype was associated with an increased risk of vitamin D insufficiency and of dyslipidemia in our study. This association with dyslipidemia has not already been reported but, mutations in *DHCR7* are associated with Smith-Lemli-Opitz Syndrome in which homozygous individuals present low serum cholesterol levels [11,28] associated with other abnormalities.

Although vitamin D deficiency has been consistently associated with obesity, some authors failed to observe an association between BMI and some genetic variants in the vitamin D pathway while they were associated with 25(OH)D levels [29-32]. According to some others, this lack of association could be explained by the fact that linear increases in serum 25(OH)D would not have a substantial influence on BMI [32]. Interestingly, our results highlighted a significant association between the rs2298849 alone and overweight (i.e. taking into account the BMI as a categorical variable) while no relationship was found between the rs2298849 genotype score and BMI. We also noted a sex difference in this relationship with women carrying the TT/TC genotypes exhibiting a higher frequency of overweight compared with those carrying the CC genotype, whereas no relationship was found in men. In a study in Caucasian nuclear families, the authors also found a female-specific association between another SNP (rs17467825) in *GC* and the percentage of fat mass [8]. This sex-specific association suggests disparities in the sensitivity to the SNP related to female (such as estrogen) or male (such as testosterone) hormones. A possible effect of sex-hormone-binding globulin should be considered. This hormone binds and transports the sex steroid hormones (mainly testosterone) in the circulation [33] and its variation may contribute to susceptibility to metabolic and cardiovascular outcomes [34]. Additionally, in a recent study in Chinese women, while the authors did not find an association between BMI and SNPs in the *GC* gene, BMI was associated with two other SNPs of the vitamin D pathway genes (rs22488359 in *CYP24A1* and rs10832313 in *CYP2R1*) [35]. This is not surprising, given that ethnic disparities in the relationships between genes and diseases have been widely reported.

The results of the linear regression of 25(OH)D showed that the genotype score based on rs2282679 in *GC* and rs12785878 in *NADSYN1* alone accounted for 3% of the variation in 25(OH)D. The addition of age, sex and BMI in the multivariate model increased the R^2^ by 5%. The variation in 25(OH)D associated with the genetic factors is not very high [13]. Other factors such as sun exposure, diet, ethnicity and season contributed more significantly to this variation [36] and may also interact with genetic effects on vitamin D status. Nevertheless, our results showing a significant average change in 25(OH)D levels of −5.5 ng/mL for a score of 3 (vs 0–1), is an important finding in our Afro-Caribbean population at risk of vitamin D insufficiency.

It should be noted that the present study had some limitations, including a small sample size although the overall population of the island is also small (400 000 inhabitants). Because of their low power, studies on small samples can lead to false positive or false negative results. In addition, the concentrations of serum 25(OH)D were examined using a single measurement for each subject. However, our study also had considerable strengths. First, all participants had high skin pigmentation and were living on an island which has annually a sunny climate. This is of importance since ethnic variation in the *GC* gene has been described [10] and seasonal variations are involved in the metabolism of vitamin D. Second, the exclusion of patients with diabetes from the study minimized the impact of insulin resistance/dependence [37] on the association between SNPs, serum 25(OH)D levels, and other risk factors. Our findings corroborated those previously reported in European and African-American individuals regarding genetic associations with vitamin D status and extended the relevant studies for a specific population sample. Third, this study is the first to analyze the effects of SNPs in the *GC* and *NADSYN1* genes concomitantly on three cardio-metabolic risk factors (overweight, dyslipidemia, and vitamin D insufficiency/deficiency).

## Conclusions

Although the role of genetic factors in the metabolism of vitamin D is recognized, much remains to be understood regarding this relationship. Our results obtained from non-diabetic Afro-Caribbean individuals confirm the associations of the *GC* and *NADSYN1* genes with vitamin D status and suggest that polymorphisms in these genes contribute to dyslipidemia and overweight independently of 25 hydroxyvitamin D levels. As vitamin D status is linked to several adverse outcomes [38], genetic variants associated with vitamin D levels may also play a role in the etiology of these diseases. Thus, it will be of interest to confirm these findings in other populations and ethnic groups.

## Abbreviations

25(OH)D: 25 hydroxyvitamin D; BMI: Body mass index; GC: Group specific component; CYP2R1: Cytochrome P450, family 2, subfamily R, polypeptide 1; NADSYN1: Nicotinamide adenine dinucleotidesynthetase 1; SNPs: Single nucleotide polymorphisms; VitD: Vitamin D; GWAS: Genome-wide association study.

## Competing interests

The authors declare that they have no competing interests.

## Authors’ contributions

LF conceived of the study and participated in its design, performed the statistical analysis and involved in writing of the manuscript. FLVC, participated in the design of the study and involved in writing of the manuscript. LL, JD, CA, CF, MLP, LLi, FB involved in writing of the manuscript. J P, JDu participated in the design and coordination of the study. All authors read and approved the final manuscript.

## Pre-publication history

The pre-publication history for this paper can be accessed here:

http://www.biomedcentral.com/1472-6823/13/36/prepub
